# Hadamard and Fejér–Hadamard inequalities for extended generalized fractional integrals involving special functions

**DOI:** 10.1186/s13660-018-1701-3

**Published:** 2018-05-18

**Authors:** Shin Min Kang, Ghulam Farid, Waqas Nazeer, Bushra Tariq

**Affiliations:** 1 Department of Mathematics and Research Institute of Natural Science, Gyeongsang National University, Jinju, Korea; 2 Center for General Education, China Medical University, Taichung, Taiwan; 3 Department of Mathematics, COMSATS University, Attock Campus, Pakistan; 4 Division of Science and Technology, University of Education, Lahore, Pakistan; 5 GGPS Kamalpur Alam, Attock, Pakistan

**Keywords:** 26A51, 26A33, 33E15, 26D15, Convex function, *m*-convex functions, Hadamard inequality, Fejér–Hadamard inequality, Fractional integrals, Extended generalized Mittag-Leffler function

## Abstract

In this paper we prove the Hadamard and the Fejér–Hadamard inequalities for the extended generalized fractional integral operator involving the extended generalized Mittag-Leffler function. The extended generalized Mittag-Leffler function includes many known special functions. We have several such inequalities corresponding to special cases of the extended generalized Mittag-Leffler function. Also there we note the known results that can be obtained.

## Introduction

A real-valued function $f:I\rightarrow\mathbb{R}$, where *I* is an interval in $\mathbb{R}$ is called convex if

$$f\bigl(\alpha x+(1-\alpha)y\bigr) \leq\alpha f(x) +(1-\alpha)f(y), $$

where $\alpha \in [0,1]$, $x, y \in I$.

Convex functions play a vital role in mathematical analysis. They have been considered for defining and finding new dimensions of analysis. In [1] Toader define the concept of *m*-convexity: an intermediate between usual convexity and star shape function.

### Definition 1.1

A function $f:[0,b]\rightarrow\mathbb{R}$, $b>0$, is said to be *m*-convex, where $m\in[0,1]$, if we have

$$ f \bigl(tx+m(1-t)y \bigr)\leq tf(x)+m(1-t)f(y) $$

for all $x,y\in[0,b]$ and $t\in[0,1]$.

If we take $m=1$, then we recapture the concept of convex functions defined on $[0,b]$, and if we take $m=0$, then we get the concept of starshaped functions defined on $[0,b]$. We recall that $f:[0,b]\to \mathbb{R}$ is called *starshaped* if

$$f(tx)\leq tf(x) \quad \mbox{for all } t\in[0,1] \mbox{ and } x\in[0,b]. $$

If we denote by $K_{m}(b)$ the set of *m*-convex functions on $[0,b]$ for which $f(0)<0$, then we have

$$K_{1}(b) \subset K_{m}(b) \subset K_{0}(b), $$

whenever $m\in(0,1)$. Note that in the class $K_{1}(b)$ there are only convex functions $f:[0,b]\to\mathbb{R}$ for which $f(0)\leq0$ (see [2]). An *m*-convex function need not be a convex function, as the following example shows.

### Example 1.1

[3] The function $f:[0,\infty)\to\mathbb{R}$, given by

$$f(x)=\frac{1}{12} \bigl(x^{4}-5x^{3}+9x^{2}-5x \bigr) $$

is a $\frac{16}{17}$-convex function but it is not an *m*-convex function for $m\in(\frac{16}{17},1]$.

For more results and inequalities related to *m*-convex functions one can consult for example [2, 4–6]. In the literature the integral inequality

1.1 $$ f \biggl(\frac{a+b}{2} \biggr)\leq\frac{1}{b-a} \int ^{b}_{a}f(x)\,dx\leq\frac{f(a)+f(b)}{2}, $$

where $f : I\rightarrow\mathbb{R}$ is a convex function on the interval *I* of real numbers and $a,b \in I$ with $a< b$, is known as the Hadamard inequality. If *f* is concave, then the above inequality holds in the reverse direction. The Hadamard inequality has always retained the attention of mathematicians and a lot of results have been produced about it, for example see [6–12] and the references cited therein.

In [13] Fejér gave a generalization of the Hadamard inequality as follows.

### Theorem 1.1

*Let* $f:[a,b]\rightarrow\mathbb{R}$ *be a convex function and* $g:[a,b]\rightarrow\mathbb{R}$ *be a non*-*negative*, *integrable and symmetric to* $\frac{a+b}{2}$. *Then the following inequality holds*:

1.2 $$ f \biggl(\frac{a+b}{2} \biggr) \int_{a}^{b}g(x)\,dx \leq \int _{a}^{b}f(x)g(x)\,dx \leq\frac{f(a)+f(b)}{2} \int_{a}^{b}g(x)\,dx. $$

In the literature inequality (1.2) is known as the Fejér–Hadamard inequality.

Nowadays the Hadamard and the Fejér–Hadamard inequalities via fractional calculus are in focus of researchers. Recently a lot of papers have been dedicated to this field (see [4, 14–16] and the references therein). Fractional calculus refers to integration or differentiation of fractional order, the origin of fractional calculus is as old as calculus. For a historical survey of this field the reader is referred to [17–21].

Fractional integral inequalities are useful in establishing the uniqueness of solutions for certain fractional partial differential equations. They also provide upper and lower bounds for the solutions of fractional boundary value problems. Many researchers have explored certain extensions and generalizations of integral inequalities by involving fractional calculus (see [14, 16, 22, 23]).

We are going to give the Hadamard and the Fejér–Hadamard inequalities for the extended generalized fractional integral operator containing the extended generalized Mittag-Leffler function [24]. We give a two sided definition of the extended generalized fractional integral operator containing the extended generalized Mittag-Leffler function as follows:

### Definition 1.2

Let $\delta,\alpha,\beta,\tau,c \in\mathbb{C}$ and $\mathbb {R}(\delta), \mathbb{R}(\alpha), \mathbb{R}(\beta), \mathbb {R}(\tau), \mathbb{R}(c)>0$, $p\geq0$ and $q,r>0$. Then the extended generalized fractional integral operator $\epsilon^{\omega,\delta,q,r,c}_{\cdot,\alpha ,\beta,\tau}$ containing the extended generalized Mittag-Leffler function $E^{\delta, r, q, c}_{\alpha,\beta, \tau}$ for a real-valued continuous function *f* is defined by

1.3 $$ \bigl(\epsilon^{\omega,\delta,q,r,c}_{a^{+},\alpha,\beta,\tau }f\bigr) (x;p)= \int_{a}^{x}(x-t)^{\beta-1}E^{\delta, r, q, c}_{\alpha ,\beta, \tau} \bigl(\omega(x-t)^{\alpha};p\bigr) f(t)\,dt, $$

and

1.4 $$ \bigl(\epsilon^{\omega,\delta,q,r,c}_{b^{-},\alpha,\beta,\tau }f\bigr) (x;p)= \int_{x}^{b}(t-x)^{\beta-1}E^{\delta, r, q, c}_{\alpha ,\beta, \tau} \bigl(\omega(t-x)^{\alpha};p\bigr) f(t)\,dt, $$

where the function $E^{\delta, r, q, c}_{\alpha,\beta, \tau}(t;p)$ is the extended generalized Mittag-Leffler function defined as

1.5 $$ E^{\delta, r, q, c}_{\alpha,\beta, \tau}(t;p)=\sum _{n=0}^{\infty }\frac{\beta_{p}(\delta+nq, c-\delta)}{\beta(\delta, c-\delta )}\frac{(c)_{nq}}{\Gamma(\alpha n+\beta)} \frac{z^{n}}{(\tau)_{nr}}, $$

where the generalized beta function $\beta_{p}(x,y)$ is defined by

1.6 $$ \beta_{p}(x,y)= \int^{1}_{0}t^{(x-1)}(1-t)^{y-1}e^{\frac{-p}{t(1-t)}} \,dt. $$

For $\omega=0$ along with $p=0$, the integral operator $\epsilon^{\omega,\delta,q,r,c}_{\cdot,\alpha,\beta,\tau}$ would correspond essentially to the two sided Riemann–Liouville fractional integral operator

$$\begin{aligned}& J_{a+}^{\beta}f(x)=\frac{1}{\Gamma(\beta)} \int_{a}^{x}(x-t)^{\beta-1}f(t)\,dt,\quad \beta>0, \\& J_{b-}^{\beta}f(x)=\frac{1}{\Gamma(\beta)} \int_{x}^{b}(t-x)^{\beta-1}f(t)\,dt,\quad \beta>0. \end{aligned}$$

In [24–29] fractional boundary value problems and fractional differential equations are studied along with properties of Mittag-Leffler function. In the following results we see some properties of the Mittag-Leffler function [24].

### Theorem 1.2

*The series in* (1.5) *is absolutely convergent for all values of* *t* *provided that* $q< r+\mathbb{R}(\alpha)$. *Moreover*, *if* $q=r+\mathbb {R}(\alpha)$, *then* $E^{\delta, r, q, c}_{\alpha,\beta, \tau}(t;p)$ *converges for* $|t|<\frac{r^{r}\mathbb{R}(\alpha)^{\mathbb{R}(\alpha )}}{q^{q}}$.

### Theorem 1.3

*If* $\alpha,\beta,\tau,\delta,c\in\mathbb{C}$, $\Re{(\alpha)},\Re {(\beta)},\Re{(\tau)}>0$, $\Re{(c)}>\Re{(\delta)}>0$ *with* $p\geq0$, $r>0$ *and* $q< r+\Re(\alpha)$, *then*

1.7 $$\begin{aligned}& E^{\delta, r, q, c}_{\alpha,\beta, \tau}(t;p)-E^{\delta, r, q, c}_{\alpha,\beta, \tau-1}(t;p)= \frac{tr}{1-t}\frac{d}{dt}E^{\delta , r, q, c}_{\alpha,\beta, \tau-1}(t;p),\quad \Re(\tau)>1; \end{aligned}$$

1.8 $$\begin{aligned}& E^{\delta, r, q, c}_{\alpha,\beta, \tau}(t;p)=\beta E^{\delta, r, q, c}_{\alpha,\beta+1, \tau}(t;p)+ \alpha t\frac{d}{dt}E^{\delta, r, q, c}_{\alpha,\beta+1, \tau}(t;p). \end{aligned}$$

We organize the paper so that in Sect. 2 we give the Hadamard and the Fejér–Hadamard inequalities via the extended generalized fractional integral operator $\epsilon^{\omega,\delta ,q,r,c}_{\cdot,\alpha,\beta,\tau}$. Also we mention the known results in particular. In Sect. 3 we extend the results of Sect. 2 via *m*-convex functions and in particular we obtain the results of Sect. 2 on a reduced domain.

## Hadamard and Fejér–Hadamard inequality for the extended generalized Mittag-Leffler function

In the following we give the Hadamard and the Fejér–Hadamard inequalities for a convex function via the extended generalized fractional integral operator containing the extended generalized Mittag-Leffler function defined in (1.3) and (1.4). We also show that these inequalities are generalizations of the Hadamard and the Fejér–Hadamard inequalities for the fractional integrals given in [15, 16, 30].

### Theorem 2.1

*Let* $f : [a,b]\rightarrow\mathbb{R}$ *be a positive function with* $0\leq a< b$ *and* $f\in L_{1}[a,b]$. *If* *f* *is a convex function on* $[a,b]$, *then the following inequality for the extended generalized fractional integral holds*:

2.1 $$\begin{aligned} f \biggl(\frac{a+b}{2} \biggr) \bigl(\epsilon^{\omega^{\prime},\delta ,q,r,c}_{a^{+},\alpha,\beta,\tau}1 \bigr) (b;p) \leq&\frac{(\epsilon ^{\omega^{\prime},\delta,q,r,c}_{a^{+},\alpha,\beta,\tau }f)(b;p)+(\epsilon^{\omega^{\prime},\delta,q,r,c}_{b^{-},\alpha ,\beta,\tau}f)(a;p)}{2} \\ \leq& \biggl(\frac{f(a)+f(b)}{2} \biggr) \bigl(\epsilon^{\omega^{\prime },\delta,q,r,c}_{b^{-},\alpha,\beta,\tau}1 \bigr) (a;p), \end{aligned}$$

*where* $\omega^{\prime}=\frac{w}{(b-a)^{\alpha}}$.

### Proof

Since *f* is a convex function on $[a,b]$, for $t\in [0,1]$ we have

2.2 $$ f \biggl(\frac{(ta+(1-t)b)+((1-t)a+tb)}{2} \biggr)\leq\frac {f(ta+(1-t)b)+f((1-t)a+tb)}{2}. $$

Multiplying both sides of the above inequality with $t^{\beta -1}E^{\delta, r, q, c}_{\alpha,\beta, \tau}(\omega t^{\alpha};p)$ we get

$$\begin{aligned} &2t^{\beta-1}E^{\delta, r, q, c}_{\alpha,\beta, \tau}\bigl(\omega t^{\alpha};p \bigr)f \biggl(\frac{a+b}{2} \biggr) \\ &\quad \leq t^{\beta -1}E^{\delta, r, q, c}_{\alpha,\beta, \tau}\bigl(\omega t^{\alpha };p\bigr) \bigl(f\bigl(ta+(1-t)b\bigr)+f\bigl((1-t)a+tb\bigr) \bigr). \end{aligned}$$

Integrating with respect to *t* over $[0,1]$ we have

$$\begin{aligned} \begin{aligned} &2f \biggl(\frac{a+b}{2} \biggr) \int^{1}_{0}{t^{\beta-1}E^{\delta, r, q, c}_{\alpha,\beta, \tau} \bigl(\omega t^{\alpha};p\bigr)}\,dt \\ &\quad \leq \int^{1}_{0}t^{\beta-1}E^{\delta, r, q, c}_{\alpha,\beta, \tau} \bigl(\omega t^{\alpha};p\bigr)f\bigl(ta+(1-t)b\bigr)\,dt \\ &\qquad {}+ \int^{1}_{0}t^{\beta -1}E^{\delta, r, q, c}_{\alpha,\beta, \tau} \bigl(\omega t^{\alpha };p\bigr)f\bigl((1-t)a+tb\bigr)\,dt. \end{aligned} \end{aligned}$$

If we put $u=at+(1-t)b$, then $t=\frac{b-u}{b-a}$, and if $v=(1-t)a+tb$, then $t=\frac{v-a}{b-a}$. So using Definition 1.2 one has

2.3 $$ f \biggl(\frac{a+b}{2} \biggr) \bigl(\epsilon^{\omega^{\prime},\delta ,q,r,c}_{a^{+},\alpha,\beta,\tau}1 \bigr) (b;p)\leq\frac{(\epsilon ^{\omega^{\prime},\delta,q,r,c}_{a^{+},\alpha,\beta,\tau }f)(b;p)+(\epsilon^{\omega^{\prime},\delta,q,r,c}_{b^{-},\alpha ,\beta,\tau}f)(a;p)}{2}. $$

Again by using the fact that *f* is a convex function on $[a,b]$ and for $t\in[0,1]$ we have

2.4 $$ f\bigl(ta+(1-t)b\bigr)+f\bigl((1-t)a+tb\bigr)\leq tf(a)+(1-t)f(b)+(1-t)f(a)+tf(b). $$

Now multiplying with $t^{\beta-1}E^{\delta, r, q, c}_{\alpha,\beta, \tau}(\omega t^{\alpha};p)$ and integrating over $[0,1]$ we get

$$\begin{aligned}& \int^{1}_{0}t^{\beta-1}E^{\delta, r, q, c}_{\alpha,\beta, \tau } \bigl(\omega t^{\alpha};p\bigr)f\bigl(ta+(1-t)b\bigr)\,dt+ \int^{1}_{0}t^{\beta -1}E^{\delta, r, q, c}_{\alpha,\beta, \tau} \bigl(\omega t^{\alpha };p\bigr)f\bigl((1-t)a+tb\bigr)\,dt \\& \quad \leq \bigl[f(a)+f(b) \bigr] \int^{1}_{0}t^{\beta-1}E^{\delta, r, q, c}_{\alpha,\beta, \tau} \bigl(\omega t^{\alpha};p\bigr)\,dt, \end{aligned}$$

from which by using a change of variables as for (2.3) and Definition 1.2 we get

2.5 $$ \bigl(\epsilon^{\omega^{\prime},\delta,q,r,c}_{a^{+},\alpha,\beta,\tau }f\bigr) (b;p)+\bigl( \epsilon^{\omega^{\prime},\delta,q,r,c}_{b^{-},\alpha ,\beta,\tau}f\bigr) (a;p)\leq \bigl(f(a)+f(b) \bigr) \bigl(\epsilon^{\omega ^{\prime},\delta,q,r,c}_{b^{-},\alpha,\beta,\tau}1\bigr) (a;p). $$

From the inequalities (2.3) and (2.5) we get the inequality in (2.1). □

In the following remark we mention some published results.

### Remark 2.1

In Theorem 2.1:

(i) if we take $p=0$, then we get [30, Theorem 2.1];
(ii) if we take $\omega=p=0$, then we get [16, Theorem 2];
(iii) if along $\omega=p=0$ we take $\alpha=1$, then we get (1.1).

In the following we give the Fejér–Hadamard inequality for the extended generalized fractional integral operator containing the extended generalized Mittag-Leffler function defined in (1.3) and (1.4).

### Theorem 2.2

*Let* $f : [a,b]\rightarrow\mathbb{R}$ *be a convex function with* $0\leq a< b$ *and* $f\in L_{1}[a,b]$. *Also*, *let* $g: [a,b]\rightarrow\mathbb{R}$ *be a function which is non*-*negative*, *integrable and symmetric about* $\frac{a+b}{2}$. *Then the following inequality for the extended generalized fractional integral holds*:

2.6 $$\begin{aligned} \begin{aligned}[b] f \biggl(\frac{a+b}{2} \biggr) \bigl(\epsilon^{\omega^{\prime},\delta ,q,r,c}_{a^{+},\alpha,\beta,\tau}g \bigr) (b;p)&\leq\frac{(\epsilon ^{\omega^{\prime},\delta,q,r,c}_{a^{+},\alpha,\beta,\tau }fg)(b;p)+(\epsilon^{\omega^{\prime},\delta,q,r,c}_{b^{-},\alpha ,\beta,\tau}fg)(a;p)}{2} \\ &\leq\frac{f(a)+f(b)}{2}\bigl(\epsilon^{\omega^{\prime},\delta ,q,r,c}_{b^{-},\alpha,\beta,\tau}g\bigr) (a;p), \end{aligned} \end{aligned}$$

*where* $\omega^{\prime}=\frac{w}{(b-a)^{\alpha}}$.

### Proof

Multiplying (2.2) with $t^{\beta-1}E^{\delta, r, q, c}_{\alpha,\beta, \tau}(\omega t^{\alpha};p)g(tb+(1-t)a)$ we get

$$\begin{aligned} &2t^{\beta-1}E^{\delta, r, q, c}_{\alpha,\beta, \tau}\bigl(\omega t^{\alpha};p \bigr)f \biggl(\frac{a+b}{2} \biggr)g\bigl(tb+(1-t)a\bigr) \\ &\quad \leq t^{\beta-1}E^{\delta, r, q, c}_{\alpha,\beta, \tau}\bigl(\omega t^{\alpha};p\bigr) \bigl(f\bigl(ta+(1-t)b\bigr)+f\bigl((1-t)a+tb\bigr) \bigr)g\bigl(tb+(1-t)a\bigr). \end{aligned}$$

Integrating with respect to *t* over $[0,1]$ we have

$$\begin{aligned} &2f \biggl(\frac{a+b}{2} \biggr) \int^{1}_{0}{t^{\beta-1}E^{\delta, r, q, c}_{\alpha,\beta, \tau} \bigl(\omega t^{\alpha};p\bigr)g\bigl(tb+(1-t)a\bigr)}\,dt \\ &\quad \leq \int^{1}_{0}t^{\beta-1}E^{\delta, r, q, c}_{\alpha,\beta, \tau} \bigl(\omega t^{\alpha};p\bigr)f\bigl(ta+(1-t)b\bigr)g\bigl(tb+(1-t)a\bigr) \,dt \\ &\qquad {}+ \int ^{1}_{0}t^{\beta-1}E^{\delta, r, q, c}_{\alpha,\beta, \tau} \bigl(\omega t^{\alpha};p\bigr)f\bigl((1-t)a+tb\bigr)g\bigl(tb+(1-t)a\bigr) \,dt. \end{aligned}$$

If we put $u=at+(1-t)b$, then $t=\frac{b-u}{b-a}$ and if $v=(1-t)a+tb$, then $t=\frac{v-a}{b-a}$. So one has

$$\begin{aligned} &2f \biggl(\frac{a+b}{2} \biggr) \int^{b}_{a}{ (b-u )^{\beta -1}E^{\delta, r, q, c}_{\alpha,\beta, \tau} \biggl(\omega \biggl(\frac{b-u}{b-a} \biggr)^{\alpha}:p \biggr)g(a+b-u)}\,du \\ &\quad \leq \int^{b}_{a} (b-u )^{\beta-1}E^{\delta, r, q, c}_{\alpha,\beta, \tau} \biggl(\omega \biggl(\frac{b-u}{b-a} \biggr)^{\alpha};p \biggr)f(u)g(a+b-u)\,du \\ &\qquad {}+ \int^{a}_{b} (v-a )^{\beta-1}E^{\delta, r, q, c}_{\alpha,\beta, \tau} \biggl(\omega \biggl(\frac{v-a}{b-a} \biggr)^{\alpha};p \biggr)f(v)g(a+b-v)\,dv. \end{aligned}$$

By the symmetry of the function *g* about $\frac{a+b}{2}$ one can see $g(a+b-x)=g(x)$, $x\in[a,b]$, therefore, using this fact and Definition 1.2, we have

2.7 $$ f \biggl(\frac{a+b}{2} \biggr) \bigl(\epsilon^{\omega^{\prime},\delta ,q,r,c}_{a^{+},\alpha,\beta,\tau}g \bigr) (b;p)\leq\frac{(\epsilon ^{\omega^{\prime},\delta,q,r,c}_{a^{+},\alpha,\beta,\tau }fg)(b;p)+(\epsilon^{\omega^{\prime},\delta,q,r,c}_{b^{-},\alpha ,\beta,\tau}fg)(a;p)}{2}. $$

Now multiplying (2.4) with $t^{\beta-1}E^{\delta, r, q, c}_{\alpha,\beta, \tau}(\omega t^{\alpha};p)g(ta+(1-t)b)$ and integrating with respect to *t* over $[0,1]$ we get

$$\begin{aligned} \begin{aligned} & \int^{1}_{0}t^{\beta-1}E^{\delta, r, q, c}_{\alpha,\beta, \tau } \bigl(\omega t^{\alpha};p\bigr)f\bigl(ta+(1-t)b\bigr)g\bigl(ta+(1-t)b\bigr) \,dt \\ &\qquad {}+ \int ^{1}_{0}t^{\beta-1}E^{\delta, r, q, c}_{\alpha,\beta, \tau} \bigl(\omega t^{\alpha};p\bigr)f\bigl((1-t)a+tb\bigr)g\bigl(ta+(1-t)b\bigr) \,dt \\ &\quad \leq \bigl(f(a)+f(b) \bigr) \int^{1}_{0}t^{\beta-1}E^{\delta, r, q, c}_{\alpha,\beta, \tau} \bigl(\omega t^{\alpha};p\bigr)g\bigl(ta+(1-t)b\bigr)\,dt. \end{aligned} \end{aligned}$$

From this by a change of variables as for (2.7) Definition 1.2 we get

2.8 $$ \bigl(\epsilon^{\omega^{\prime},\delta,q,r,c}_{a^{+},\alpha,\beta,\tau }fg\bigr) (b;p)+\bigl( \epsilon^{\omega^{\prime},\delta,q,r,c}_{b^{-},\alpha ,\beta,\tau}fg\bigr) (a;p)\leq \bigl(f(a)+f(b) \bigr) \bigl(\epsilon^{\omega ^{\prime},\delta,q,r,c}_{b^{-},\alpha,\beta,\tau}g\bigr) (a;p). $$

From inequalities (2.8) and (2.7) we get the inequality in (2.6). □

In the following we mention some published results.

### Remark 2.2

In Theorem 2.2:

(i) if we take $g=1$, then we get Theorem 2.1;
(ii) if we take $p=0$, then we get [30, Theorem 2.2];
(iii) if we take $\omega=p=0$, then we get [15, Theorem 2.2].

## Hadamard and Fejér–Hadamard inequality for *m*-convex function via the extended generalized Mittag-Leffler function

In the following we give the Hadamard and the Fejér–Hadamard inequalities for an *m*-convex function via the extended generalized fractional integral operator containing the extended generalized Mittag-Leffler function defined in (1.3) and (1.4). We also show that these inequalities are generalizations of the Hadamard and the Fejér–Hadamard inequalities for the fractional integrals given in [4, 15, 16, 31].

### Theorem 3.1

*Let* $f : [0,\infty)\rightarrow\mathbb{R}$ *be a positive function*. *Let* $a,b\in[0,\infty)$ *with* $0\leq a< mb$ *and* $f\in L_{1}[a,mb]$. *If* *f* *is* *m*-*convex function on* $[a,mb]$, *then the following inequality for the extended generalized fractional integral holds*:

3.1 $$\begin{aligned} &f \biggl(\frac{a+mb}{2} \biggr) \bigl(\epsilon^{\omega^{\prime},\delta ,q,r,c}_{a^{+},\alpha,\beta,\tau}1 \bigr) (mb;p) \\ &\quad \leq\frac{(\epsilon ^{\omega^{\prime},\delta,q,r,c}_{a^{+},\alpha,\beta,\tau }f)(mb;p)+m^{\beta+1}(\epsilon^{m^{\alpha}\omega^{\prime},\delta ,q,r,c}_{b^{-},\alpha,\beta,\tau}f)(\frac{a}{m};p)}{2} \\ &\quad \leq\frac{m^{\beta+1}}{2}\biggl[\frac{f(a)-m^{2}f (\frac {a}{m^{2}} )}{mb-a}\bigl( \epsilon^{m^{\alpha}\omega^{\prime},\delta ,q,r,c}_{b^{-},\alpha,\beta+1,\tau}1\bigr) \biggl(\frac{a}{m};p \biggr) \\ &\qquad {}+ \biggl(f(b)+mf \biggl(\frac{a}{m^{2}} \biggr) \biggr) \bigl( \epsilon^{m^{\alpha}\omega^{\prime},\delta ,q,r,c}_{b^{-},\alpha,\beta,\tau}1\bigr) \biggl(\frac{a}{m};p \biggr) \biggr], \end{aligned}$$

*where* $\omega^{\prime}=\frac{w}{(mb-a)^{\alpha}}$.

### Proof

Since *f* is an *m*-convex function on $[a,mb]$, for $t\in[0,1]$ we have

3.2 $$\begin{aligned} &f \biggl(\frac{(ta+m(1-t)b)+m ((1-t)\frac{a}{m}+tb )}{2} \biggr) \\ &\quad \leq\frac{f(ta+m(1-t)b)+mf ((1-t)\frac{a}{m}+tb )}{2}. \end{aligned}$$

Multiplying with $t^{\beta-1}E^{\delta, r, q, c}_{\alpha,\beta, \tau}(\omega t^{\alpha};p)$ both sides of the above inequality we get

$$\begin{aligned} \begin{aligned} &2t^{\beta-1}E^{\delta, r, q, c}_{\alpha,\beta, \tau}\bigl(\omega t^{\alpha};p \bigr)f \biggl(\frac{a+mb}{2} \biggr) \\ &\quad \leq t^{\beta-1}E^{\delta, r, q, c}_{\alpha,\beta, \tau}\bigl(\omega t^{\alpha };p\bigr) \biggl(f\bigl(ta+m(1-t)b\bigr)+mf \biggl((1-t)\frac{a}{m}+tb \biggr) \biggr). \end{aligned} \end{aligned}$$

Integrating with respect to *t* over $[0,1]$ we have

$$\begin{aligned} &2f \biggl(\frac{a+mb}{2} \biggr) \int^{1}_{0}{t^{\beta-1}E^{\delta, r, q, c}_{\alpha,\beta, \tau} \bigl(\omega t^{\alpha};p\bigr)}\,dt \\ &\quad \leq \int^{1}_{0}t^{\beta-1}E^{\delta, r, q, c}_{\alpha,\beta, \tau} \bigl(\omega t^{\alpha};p\bigr)f\bigl(ta+m(1-t)b\bigr)\,dt \\ &\qquad {}+m \int^{1}_{0}t^{\beta -1}E^{\delta, r, q, c}_{\alpha,\beta, \tau} \bigl(\omega t^{\alpha };p\bigr)f \biggl((1-t)\frac{a}{m}+tb \biggr) \,dt. \end{aligned}$$

If we put $u=at+m(1-t)b$, then $t=\frac{mb-u}{mb-a}$ and if $v=(1-t)\frac{a}{m}+tb$, then $t=\frac{v-\frac{a}{m}}{b-\frac {a}{m}}$. So by Definition 1.2 one has

3.3 $$\begin{aligned}& f \biggl(\frac{a+mb}{2} \biggr) \bigl(\epsilon^{\omega^{\prime},\delta ,q,r,c}_{a^{+},\alpha,\beta,\tau}1 \bigr) (mb;p) \\& \quad \leq\frac{(\epsilon ^{\omega^{\prime},\delta,q,r,c}_{a^{+},\alpha,\beta,\tau }f)(mb;p)+m^{\beta+1}(\epsilon^{m^{\alpha}\omega^{\prime},\delta ,q,r,c}_{b^{-},\alpha,\beta,\tau}f) (\frac{a}{m};p )}{2}. \end{aligned}$$

Again by using that *f* is an *m*-convex function we have

3.4 $$\begin{aligned} &f\bigl(ta+m(1-t)b\bigr)+mf \biggl((1-t)\frac{a}{m}+tb \biggr) \\ &\quad \leq tf(a)+m(1-t)f(b)+m^{2}(1-t)f \biggl(\frac{a}{m^{2}} \biggr)+mtf(b). \end{aligned}$$

Now multiplying with $t^{\beta-1}E^{\delta, r, q, c}_{\alpha,\beta, \tau}(\omega t^{\alpha};p)$ and integrating with respect to *t* over $[0,1]$ we get

$$\begin{aligned} & \int^{1}_{0}t^{\beta-1}E^{\delta, r, q, c}_{\alpha,\beta, \tau } \bigl(\omega t^{\alpha};p\bigr)f\bigl(ta+m(1-t)b\bigr)\,dt \\ &\qquad {}+m \int^{1}_{0}t^{\beta -1}E^{\delta, r, q, c}_{\alpha,\beta, \tau} \bigl(\omega t^{\alpha };p\bigr)f \biggl((1-t)\frac{a}{m}+tb \biggr) \,dt \\ &\quad \leq \biggl[f(a)-m^{2}f \biggl(\frac{a}{m^{2}} \biggr) \biggr] \int ^{1}_{0}t^{\beta}E^{\delta, r, q, c}_{\alpha,\beta, \tau} \bigl(\omega t^{\alpha};p\bigr)\,dt \\ &\qquad {}+m \biggl[f(b)+mf \biggl(\frac{a}{m^{2}} \biggr) \biggr] \int^{1}_{0}t^{\beta-1}E^{\delta, r, q, c}_{\alpha,\beta, \tau } \bigl(\omega t^{\alpha};p\bigr)\,dt. \end{aligned}$$

From this by using a change of variables as for (3.3) and Definition 1.2 we get

3.5 $$\begin{aligned} \begin{aligned}[b] &\frac{(\epsilon^{\omega^{\prime},\delta,q,r,c}_{a^{+},\alpha ,\beta,\tau}f)(mb;p)+m^{\beta+1}(\epsilon^{m^{\alpha}\omega ^{\prime},\delta,q,r,c}_{b^{-},\alpha,\beta,\tau}f)(\frac {a}{m};p)}{2} \\ &\quad \leq\frac{m^{\beta+1}}{2}\biggl[\frac {f(a)-m^{2}f (\frac{a}{m^{2}} )}{mb-a}\bigl( \epsilon^{m^{\alpha }\omega^{\prime},\delta,q,r,c}_{b^{-},\alpha,\beta+1,\tau}1\bigr) \biggl(\frac{a}{m};p \biggr) \\ &\qquad {}+ \biggl(f(b)+mf \biggl(\frac{a}{m^{2}} \biggr) \biggr) \bigl( \epsilon^{m^{\alpha}\omega^{\prime },\delta,q,r,c}_{b^{-},\alpha,\beta,\tau}1\bigr) \biggl(\frac {a}{m};p \biggr) \biggr]. \end{aligned} \end{aligned}$$

From inequalities (3.3) and (3.5) we get the inequality in (3.1). □

In the following remark we mention some published results.

### Remark 3.1

In Theorem 3.1:

(i) if we take $p=0$, then we get [31, Theorem 3];
(ii) if we take $\omega=p=0$, then we get [4, Theorem 2.1];
(iii) if along with $\omega=p=0$, $m=1$, then we get [16, Theorem 2];
(iv) if we take $\omega=p=0$ along with $\alpha=m=1$, then we get (1.1);
(v) if we take $m=1$, then the inequality (3.1) gives the inequality (2.1) of Theorem 2.1 on the domain of *f* as $[0,b]$.

In the following we give the Fejér–Hadamard inequality for an *m*-convex function via the extended generalized fractional integral operator defined in (1.3) and (1.4).

### Theorem 3.2

*Let* $f : [0,\infty)\rightarrow\mathbb{R}$ *be a* *m*-*convex function*, $a,b\in[0,\infty)$ *with* $0\leq a< mb$ *and* $f\in L_{1}[a,mb]$. *Also*, *let* $g: [a,mb]\rightarrow\mathbb{R}$ *be a function which is non*-*negative and integrable on* $[a,mb]$. *If* $f(a+mb-mx)=f(x)$, *then the following inequality for an extended generalized fractional integral holds*:

3.6 $$\begin{aligned} &f \biggl(\frac{a+mb}{2} \biggr) \bigl(\epsilon^{\omega^{\prime},\delta ,q,r,c}_{b^{-},\alpha,\beta,\tau}g \bigr) \biggl(\frac{a}{m};p \biggr) \\ &\quad \leq\frac{(1+m)}{2}\bigl(\epsilon^{\omega^{\prime},\delta ,q,r,c}_{b^{-},\alpha,\beta+1,\tau}fg \bigr) \biggl(\frac{a}{m};p \biggr) \\ &\quad \leq\frac{1}{2}\biggl[\frac{f(a)-m^{2}f (\frac {a}{m^{2}} )}{mb-a}\bigl( \epsilon^{\omega^{\prime},\delta ,q,r,c}_{b^{-},\alpha,\beta+1,\tau}g\bigr) \biggl(\frac{a}{m};p \biggr) \\ &\qquad {}+m \biggl(f(b)+mf \biggl(\frac {a}{m^{2}} \biggr) \biggr) \bigl( \epsilon^{\omega^{\prime},\delta ,q,r,c}_{b^{-},\alpha,\beta,\tau}g\bigr) \biggl(\frac{a}{m};p \biggr) \biggr], \end{aligned}$$

*where* $\omega^{\prime}=\frac{w}{(b-\frac{a}{m})^{\alpha}}$.

### Proof

Multiplying (3.2) with $t^{\beta-1}E^{\delta, r, q, c}_{\alpha,\beta, \tau}(\omega t^{\alpha};p)g ((1-t)\frac {a}{m}+tb )$ we get

$$\begin{aligned} \begin{aligned} &2t^{\beta-1}E^{\delta, r, q, c}_{\alpha,\beta, \tau}\bigl(\omega t^{\alpha};p \bigr)f \biggl(\frac{a+mb}{2} \biggr)g \biggl((1-t)\frac {a}{m}+tb \biggr) \\ &\quad \leq t^{\beta-1}E^{\delta, r, q, c}_{\alpha ,\beta, \tau}\bigl(\omega t^{\alpha};p\bigr) \biggl(f\bigl(ta+m(1-t)b\bigr) \\ &\qquad {}+mf \biggl((1-t)\frac{a}{m}+tb \biggr)\biggr)g \biggl((1-t) \frac{a}{m}+tb \biggr). \end{aligned} \end{aligned}$$

Integrating with respect to *t* over $[0,1]$ we have

3.7 $$\begin{aligned} &2f \biggl(\frac{a+mb}{2} \biggr) \int^{1}_{0}{t^{\beta-1}E^{\delta, r, q, c}_{\alpha,\beta, \tau} \bigl(\omega t^{\alpha};p\bigr)g \biggl((1-t)\frac{a}{m}+tb \biggr)} \,dt \\ &\quad \leq \int^{1}_{0}t^{\beta-1}E^{\delta, r, q, c}_{\alpha,\beta, \tau} \bigl(\omega t^{\alpha};p\bigr)f\bigl(ta+m(1-t)b\bigr)g \biggl((1-t) \frac {a}{m}+tb \biggr)\,dt \\ &\qquad {}+m \int^{1}_{0}t^{\beta-1}E^{\delta, r, q, c}_{\alpha,\beta, \tau} \bigl(\omega t^{\alpha};p\bigr)f \biggl((1-t)\frac{a}{m}+tb \biggr)g \biggl((1-t)\frac{a}{m}+tb \biggr)\,dt. \end{aligned}$$

Setting $x=(1-t)\frac{a}{m}+tb$ and using $f(a+mb-mx)=f(x)$ along with Definition 1.2 we get

3.8 $$ f \biggl(\frac{a+mb}{2} \biggr) \bigl(\epsilon^{\omega^{\prime},\delta ,q,r,c}_{b^{-},\alpha,\beta,\tau}g \bigr) \biggl(\frac{a}{m};p \biggr)\leq (1+m) \bigl(\epsilon^{\omega^{\prime},\delta,q,r,c}_{b^{-},\alpha,\beta ,\tau}fg \bigr) \biggl(\frac{a}{m};p \biggr). $$

Now multiplying (3.4) with $t^{\beta-1}E^{\delta, r, q, c}_{\alpha,\beta, \tau}(\omega t^{\alpha};p)g ((1-t)\frac {a}{m}+tb )$ and integrating with respect to *t* over $[0,1]$ we get

$$\begin{aligned} & \int^{1}_{0}t^{\beta-1}E^{\delta, r, q, c}_{\alpha,\beta, \tau } \bigl(\omega t^{\alpha};p\bigr)f\bigl(ta+m(1-t)b\bigr)g \biggl((1-t) \frac{a}{m}+tb \biggr)\,dt \\ &\qquad {}+m \int^{1}_{0}t^{\beta-1}E^{\delta, r, q, c}_{\alpha,\beta, \tau} \bigl(\omega t^{\alpha};p\bigr)f \biggl((1-t)\frac{a}{m}+tb \biggr)g \biggl((1-t)\frac{a}{m}+tb \biggr)\,dt \\ &\quad \leq \biggl[f(a)-m^{2}f \biggl(\frac{a}{m^{2}} \biggr) \biggr] \int ^{1}_{0}t^{\beta}E^{\delta, r, q, c}_{\alpha,\beta, \tau} \bigl(\omega t^{\alpha};p\bigr)g \biggl((1-t)\frac{a}{m}+tb \biggr) \,dt \\ &\qquad {}+m \biggl[f(b)+mf \biggl(\frac{a}{m^{2}} \biggr) \biggr] \int^{1}_{0}t^{\beta -1}E^{\delta, r, q, c}_{\alpha,\beta, \tau} \bigl(\omega t^{\alpha };p\bigr)g \biggl((1-t)\frac{a}{m}+tb \biggr) \,dt. \end{aligned}$$

From this by setting $x=(1-t)\frac{a}{m}+tb$ and using $f(a+mb-mx)=f(x)$ it can be seen

3.9 $$\begin{aligned} &\frac{(1+m)}{2}\bigl(\epsilon^{\omega^{\prime},\delta ,q,r,c}_{b^{-},\alpha,\beta+1,\tau}fg\bigr) \biggl( \frac{a}{m} \biggr) \\ &\quad \leq\frac{1}{2}\biggl[\frac{f(a)-m^{2}f (\frac{a}{m^{2}} )}{mb-a}\bigl( \epsilon^{\omega^{\prime},\delta,q,r,c}_{b^{-},\alpha ,\beta+1,\tau}g\bigr) \biggl(\frac{a}{m} \biggr) \\ &\qquad {}+m \biggl(f(b)+mf \biggl(\frac{a}{m^{2}} \biggr) \biggr) \bigl( \epsilon ^{\omega^{\prime},\delta,q,r,c}_{b^{-},\alpha,\beta,\tau}g\bigr) \biggl(\frac{a}{m} \biggr) \biggr]. \end{aligned}$$

From inequalities (3.8) and (3.9) we get the inequality in (3.6). □

### Remark 3.2

In Theorem 3.2:

(i) if we take $g=1$, then we get Theorem 3.1;
(ii) if we take $g=1$, $m=1$, then we get Theorem 2.1 on the domain of *f* as $[0,b]$;
(iii) if we take $\omega=p=0$ along with $m=1$, then we get [15, Theorem 2.1].
